# Yu Ping Feng San reverses cisplatin-induced multi-drug resistance in lung cancer cells via regulating drug transporters and p62/TRAF6 signalling

**DOI:** 10.1038/srep31926

**Published:** 2016-08-25

**Authors:** Jian-Shu Lou, Lu Yan, Cathy W. C. Bi, Gallant K. L. Chan, Qi-Yun Wu, Yun-Le Liu, Yun Huang, Ping Yao, Crystal Y. Q. Du, Tina T. X. Dong, Karl W. K. Tsim

**Affiliations:** 1 Division of Life Science, Center for Chinese Medicine, The Hong Kong University of Science and Technology, Clear Water Bay, Hong Kong, China; 2 Shenzhen Research Institute, The Hong Kong University of Science and Technology, Shenzhen, 518057, China; 3 Department of Biology, Hanshan Normal University, Chaozhou, Guangdong 521041, China

## Abstract

Yu Ping Feng San (YPFS), an ancient Chinese herbal decoction composed of Astragali Radix, Atractylodis Macrocephalae Rhizoma and Saposhnikoviae Radix, has been used in the clinic for treating immune deficiency. In cancer therapy, YPFS is being combined with chemotherapy drugs to achieve improved efficacy; however, scientific evidence to illustrate this combination effect is lacking. The present study aims to demonstrate the anti-drug resistance of YPFS in cisplatin (DDP)-resistant non-small cell lung cancer cells (A549/DDP). The application of YPFS exhibited a synergistic enhancement of DDP-induced cytotoxicity as well as of the apoptotic signalling molecules. DDP-induced expression of the multi-drug-resistance efflux transporters was markedly reduced in the presence of YPFS, resulting in a higher intracellular concentration of DDP. In addition, the application of YPFS increased DDP-induced ROS accumulation and MMP depletion, decreased p62/TRAF6 signalling in DDP-treated A549/DDP cells. The co-treatment of DDP and YPFS in tumour-bearing mice reduced the tumour size robustly (by more than 80%), which was much better than the effect of DDP alone. These results indicate that YPFS can notably improve the DDP-suppressed cancer effect, which may be a consequence of the elevation of intracellular DDP via the drug transporters as well as the down regulation of p62/TRAF6 signalling.

Lung cancer is the leading cause of cancer-related deaths worldwide. As estimated by the International Agency for Research on Cancer (IACR), the number of deaths caused by lung cancer will raise to 10 million deaths per year by 2030. Almost 80% of bronchogenic carcinomas are non-small cell lung cancers (NSCLC), and approximately half of the patients that have been newly diagnosed with NSCLC will develop metastatic disease^1^. Treatment of NSCLC has been significantly improved by the discovery of epidermal growth factor receptor (EGFR) tyrosine kinase inhibitors; however, the effectiveness of these inhibitors is highly related to the EGFR genotype of the patient^2^. EGFR inhibitors induce apoptotic cell death (caspase-dependent) in lung cancer cells that express mutant EGFR but have a poor effect in cells that express wide-type EGFR^3^,^4^. Moreover, EGFR inhibitors have a poor efficacy in patients with advanced lung cancer, which accounts for more than half of the lung cancer patients^5^. Thus, platinum-based chemotherapy remains the standard first-line treatment^6^. Cisplatin (*cis*-diamminedichloroplatinum (II); DDP), a commonly used platinum-based chemotherapy drug for the treatment of lung cancer, induces cell death via caspase-dependent or independent pathways regardless of the EGFR genotype^7^.

The key flaw of treating lung cancer with DDP is the development of acquired drug resistance, resulting in reduced therapeutic efficacy^8^. During the last three decades, an intense wave of clinical and preclinical research has been aimed at the development of strategies to restore the sensitivity of NSCLC patients to DDP. Drug combination is one effective way to address this issue; a compound that is combined with DDP to increase its effect is referred to as a chemosensitizer. However, the majority of chemosensitizers failed to improve the therapeutic profile of DDP in randomized clinical trials^9^. Thus, the focus today has shifted to Traditional Chinese medicine (TCM), which has been previously used as a common way to improve the efficacy as well as to reduce the toxicity of chemotherapy for the treatment of NSCLC. Additionally, the application of TCM during chemotherapy could have an effect in reversing the drug resistance generated by anti-cancer drugs. “Yu Ping Feng San” (YPFS), an ancient TCM formula that was designed by Zhu Danxi in 1347 A.D., is composed of Astragali Radix (AR; Huangqi; the root of *Astragalus membranaceus* (Fisch.) Bunge or *Astragalus membranaceus* (Fisch.) Bunge var. *mongholicus* (Bunge) P.K. Hsiao), Atractylodis Macrocephalae Rhizoma (AMR; Baizhu; the rhizomes of *Atractylodes macrocephala* Koidz.) and Saposhnikoviae Radix (SR; Fangfeng; the roots of *Saposhnikovia divaricata* ((Turcz.) Schischk.) in a weight ratio of 1:2:1. Traditionally, YPFS is prescribed for the treatment of flus, as well as inflammation-associated diseases. YPFS was reported to increase immune function and to regulate haematopoiesis^10^,^11^. In cancer therapy, treatment with YPFS when combined with DDP showed a synergistic effect on the immune responses of hepatocarcinoma-bearing nude mice^12^. The co-treatment of YPFS and DDP could also improve the curative effects of leukopenia during chemotherapy^13^. Moreover, the application of YPFS in cultured Caco-2 monolayer cells inhibited the efflux transport of flavonoids, suggesting a possible anti-multi-drug resistance of YPFS in drug transport^14^. Here, we hypothesized that YPFS could reverse DDP-resistance in the human lung cancer cell line A549/DDP, and we subsequently elucidated the mechanism of this YPFS-mediated drug resistance.

## Results

### YPFS reverses DDP resistance in A549/DDP cells

AR, AMR and SR were boiled together in water under moderate heating conditions to generate the herbal decoction of YPFS. The final extraction was approximately 51.06 ± 3.08% (*n* = 3). To chemically standardize YPFS, the rapid resolution liquid chromatography/tandem mass spectrometry (RRLC-QQQ-MS/MS) chromatograms of YPFS were developed^11^, and these chromatograms were used to ensure the detection of the chosen chemical markers from the herbal extracts (Supplementary Fig. 1). Importantly, the chromatograms served as an index for the identification of YPFS. Fifteen chemical markers were determined in the YPFS extract defining the minimum requirement for 1 g of dried powder of standardized YPFS, i.e., calycosin-7-O-β-D-glucoside (0.339 mg/g); calycosin (0.228 mg/g); ononin (0.618 mg/g); formononetin (0.250 mg/g); astragaloside IV (0.109 mg/g); astragaloside III (0.100 mg/g); astragaloside II (0.046 mg/g); atractylenolide I (0.032 mg/g); atractylenolide II (0.043 mg/g); atractylenolide III (0.303 mg/g); prim-O-glucosylcimifugin (0.682 mg/g); 5-O-methylvisammioside (0.455 mg/g); and coumarins: scopoletin (0.005 mg/g), isopsoralen (0.005 mg/g), and psoralen (0.005 mg/g). The analytical result of YPFS was consistent with previous studies in meeting the quality requirements^11^. This formulation of YPFS, written in 1347 A.D. by Zhu Danxi, was studied instead of other formulas because this formulation provided better solubilities of the active ingredients (Supplementary Fig. 2). Moreover, this YPFS formula is the most commonly used in clinical applications.

**Figure 1:**
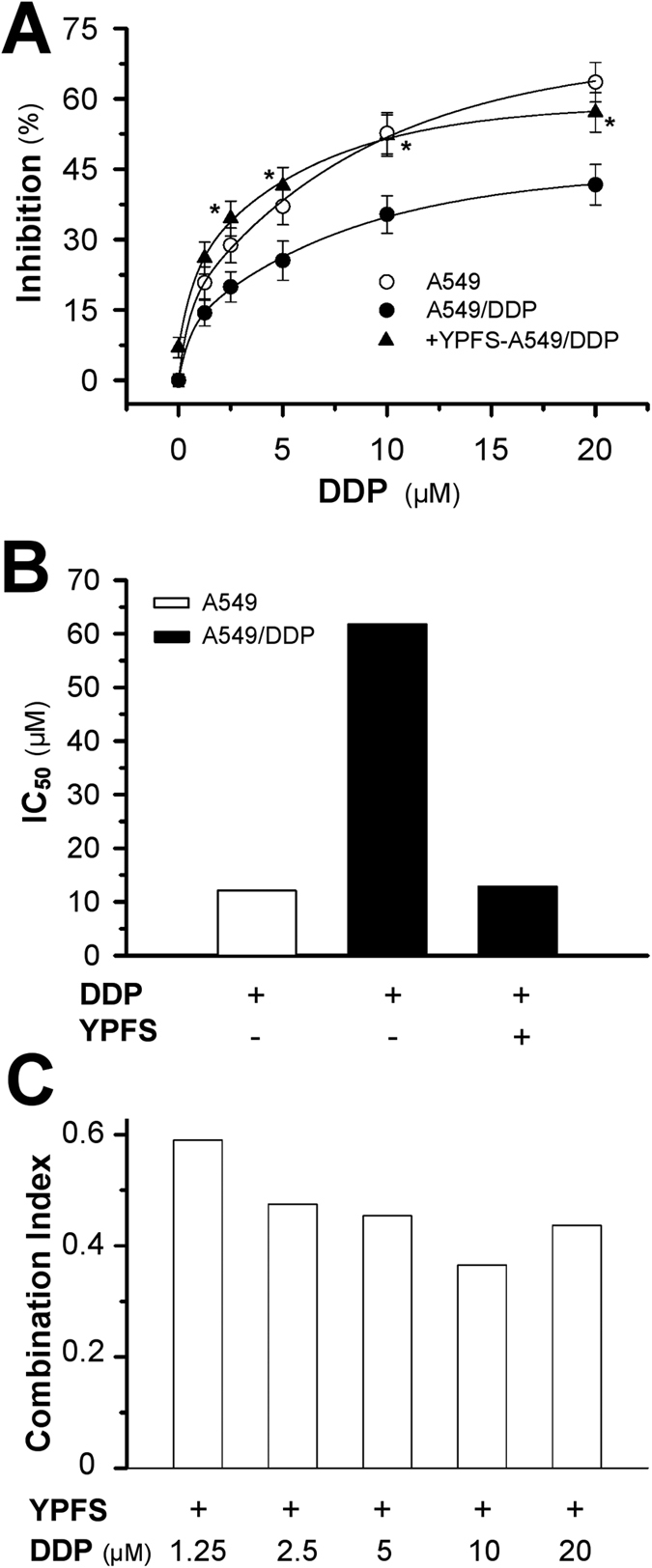
Synergetic effect of YPFS on DDP-induced A549/DDP cell death. (**A**) Cells were seeded in 96-well plates (3 × 10^3^ cells/well) were allowed to adhere overnight and subsequently were treated with increasing concentration of DDP combined with YPFS (1 mg/mL) for 48 hours. Values are in percentage of cell growth inhibition. (**B**) The IC_50_ value of DDP on A549 and A549/DDP cells, as well as in the absence and presence of YPFS (1 mg/mL). (**C**) The Combination Index (CI) was calculated using CompuSyn software. CI values <1, 1 and >1 indicate synergism, additive effect and antagonism, respectively. Each point represents the mean ± SEM, *n* = 3. *p < 0.05 versus DDP-treated alone on A549/DDP cells.

Compared to the parental A549 cells, A549/DDP cells showed drug resistance to the DDP challenge. The application of DDP inhibited cell proliferation in a dose-dependent manner; the maximum inhibition rate of ~40% was achieved at 20 μM DDP treatment for 48 hours in A549/DDP cells (Fig. 1A). The IC_50_ of DDP in A549/DDP cells was 61.8 μM, which was 4.8-fold higher than the IC_50_ of DDP in A549 cells (Fig. 1A,B). Pre-treatment with YPFS at 1 mg/mL for 12 hours before challenging with DDP enhanced the DDP-inhibited cell growth; the maximal cell inhibition was increased more than 50% by 20 μM DDP. One mg/mL of YPFS was used as the routine concentration because this dose did not affect the cell growth and had the maximum effect on the mRNAs that encode for various transporters (Supplementary Fig. 3). The effective DDP concentration for achieving ~25% inhibition was decreased from 5 μM to 1.25 μM in the presence of YPFS (Fig. 1A). In addition, the IC_50_ of DDP combined with YPFS was similar to that of DDP single use in A549 (12.8 μM), which indicated that YPFS at 1 mg/ml can sensitize DDP induced toxicity in A549/DDP (Fig. 1B). To search the possible synergistic role of YPFS in DDP-inhibited cell growth, the combination index (CI) at different DDP concentrations, combined with YFPS at 1 mg/mL, was determined: all of them were below 0.6. The lowest CI value was identified at 10 μM DDP (Fig. 1C). The result indicated that YPFS could synergistically suppress the cell proliferation, and which could reverse the DDP-induced resistance on A549/DDP cell line, i.e. showing increase sensitivity to DDP challenge.

### YPFS enhances the DDP-induced apoptosis

**Figure 2:**
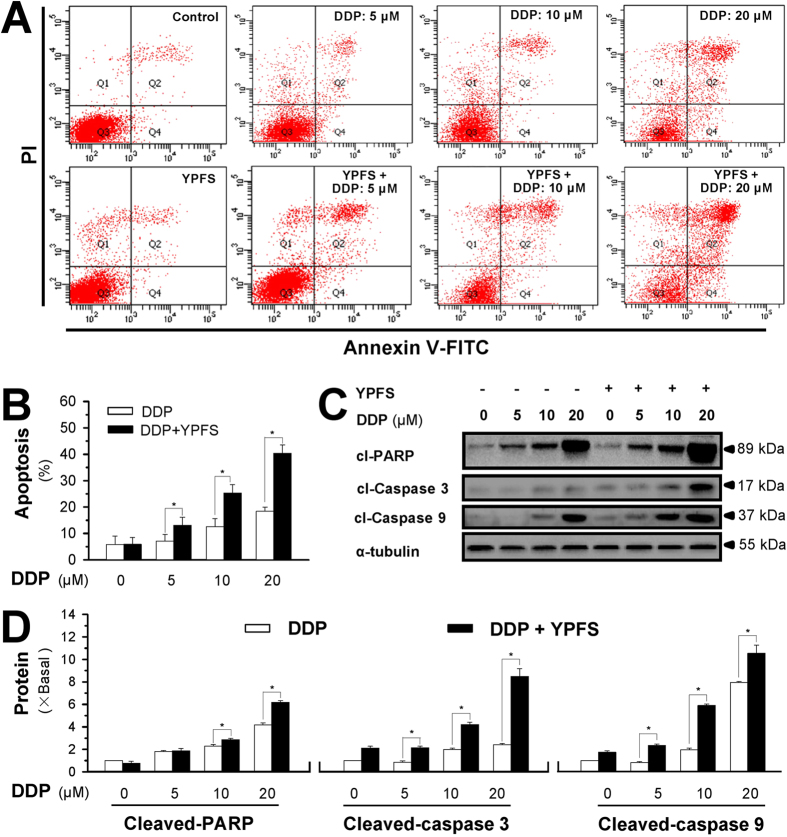
YPFS enhances DDP-induced apoptosis in cultured A549/DDP cells. (**A**) Cultured A549/DDP cells were treated with DDP at different doses for 48 hours in the presence or absence of YPFS (1 mg/mL; 12 hours of pre-treatment). Cell apoptosis was detected by flow cytometry with Annexin V/PI apoptosis detection kit. The dual parametric dot plots combining annexin V-FITC and PI fluorescence showed the viable cell population in the bottom left quadrant (Q3), the early apoptotic cells in the bottom right quadrant (Q4), and the late apoptotic cells in the top right quadrant (Q2). (**B**) Determination of apoptotic rates, as calibrated from (A). Values are in percentage of apoptotic cell number. (**C**) Western blot analyses of cleaved (cI)-caspase 3 at ~17 kDa, cleaved (cI)-caspase 9 at ~37 kDa, and cleaved (cI)-PARP at ~89 kDa. Expression of α-tubulin (~55 kDa) served as a control. The full length bot of cl-PARP is supplied in supplementary data Fig. 6. (**D**) Quantitation of cI-caspase 3, cI-caspase 9, and cI-PARP, as calibrated from (**C**). Values are in fold of change (X Basal) to control (no drug treatment). Each point represents the mean ± SEM, *n* = 3. *p < 0.05 versus DDP-treated alone.

The induction of cell apoptosis was analysed in A549/DDP cells treated with DDP in the presence and absence of YPFS. Annexin V-FITC- and PI-labelled A549/DDP cells were subjected to flow cytometry analysis, and the cells underwent apoptosis after DDP treatment in a dose-dependent manner (Fig. 2A,B). The pre-treatment of YPFS significantly increased the DDP-induced cell apoptosis. Fig. 2B shows the quantitation result, as revealed by flow cytometry analysis. In the presence of YPFS, the DDP-induced cell apoptosis was increased by greater than 50%. To further confirm these findings, western blot assays were conducted to investigate the expression of apoptotic markers, including cleaved-PARP, cleaved-caspase 3 and cleaved-caspase 9. Significant increases of cleaved-PARP, cleaved-caspase 3 and cleaved-caspase 9 were detected following co-treatment with DDP and YPFS (Fig. 2C,D).

### YPFS increases intracellular DDP

**Figure 3:**
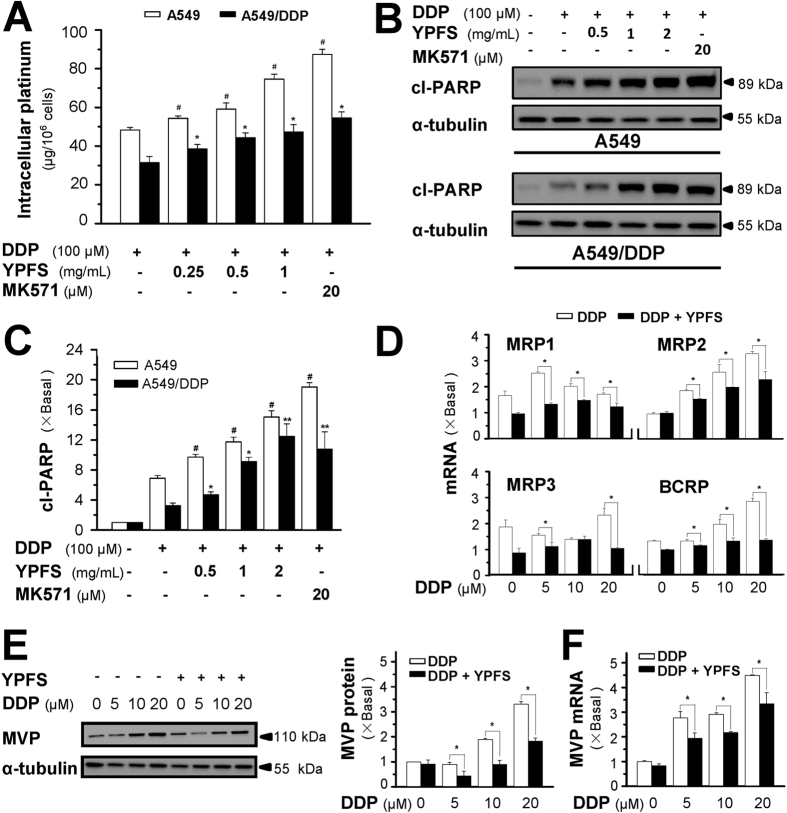
YPFS increases intracellular concentration of DDP and suppresses DDP-induced expression of drug resistant-related transporters. (**A**) Intracellular platinum (as of DDP in μg/one million cells) was measured with ICP-OES. DDP at 100 μM, in the absence or presence of YPFS at 1 mg/mL, was applied onto A549 and A549/DDP cells for 6 hours, then the cell lysate was collected for platinum determination. MK571, a MRP1 inhibitor, served as a control. (**B**) The cell treatment was done as in (**A**), the amount of cl-PARP was determined by western blot. Expression of α-tubulin (~55 kDa) served as a control. (**C**) Quantitation of cl-PARP expression. (**D**) Cultured A549/DDP cells were treated with or without YPFS (1 mg/mL) for 12 hours, followed by 48 hours of DDP treatment at various concentrations. The amounts of mRNAs encoding MRP1, MRP2, MRP3 and BCRP were determined by real-time PCR. (**E**) The cell treatment was done as in (**D**). The amount of MVP protein was determined by western blotting (left panel). Expression of α-tubulin (~55 kDa) served as a control. Protein quantitation was done (right panel). (**F**) Quantitation of MVP mRNA expression. Treatment was as that in (**D**), and real-time PCR was performed for measurement of MVP mRNA. Values are in fold of change (X Basal) to control (no drug treatment). Each point represents the mean ± SEM, *n* = 3. *p < 0.05, **p < 0.01 versus DDP-treated alone in A549/DDP, ^#^*p* < 0.05 versus DDP-treated alone in A549.

The enhancement of DDP-induced cell death by YPFS could result from blocking the multi-drug resistance of A549/DDP cells, i.e., by increasing the drug concentration inside the cell. Cultured A549/DDP cells are able to efflux DDP, which increases cell survival. Under a short exposure of DDP, the intracellular concentration of DDP, detected by ICP-OES, was higher in the A549 cells than in the A549/DDP cells (Fig. 3A). In both cell types, pre-treatment with YPFS increased the intracellular concentration of DDP in a dose-dependent manner, which was similar to MK571 treatment (Fig. 3A). In parallel, the expression of the apoptotic marker cleaved-PARP in the non-resistance cells was approximately 2-fold higher than that in the resistance cells when exposed to DDP at 100 μM for 6 hours (Fig. 3B,C). Similarly, this phenomenon was attenuated by the combination of YPFS and DDP in both A549 and A549/DDP cells (Fig. 3B,C). Application of YPFS at 1 mg/mL can sensitize the A549/DDP to express the same level of cleaved-PARP as that in A549 exposed to DDP single use. A similar blockage of apoptosis was detected with the co-treatment of DDP and MK571, an inhibitor of multi-drug resistance-associated protein (MRP1). The drug resistance of DDP in A549/DDP cells could be caused by the reduction of drug accumulation in the tumour cells, thus, YPFS reversed this DDP-mediated drug reduction. The role of YPFS in increasing the DDP concentration in A549/DDP cells could be twofold: (i) blocking the activity of transporters and (ii) decreasing the amount of transporters. The increase of intracellular DDP after a short exposure to YPFS indicated blockage of the transporter activities, and indeed, this was illustrated in another cell line^11^. To investigate the cause of variation of the transporter number, the members of the ABC family responsible for the DDP efflux were identified. mRNAs encoding MRP1, MRP2, MRP3 and breast cancer resistance protein (BCRP) were significantly induced in the presence of DDP. In all cases, pre-treatment with YPFS in A549/DDP cells markedly reduced the expression of MRP1, MRP2, MRP3 and BCRP; the reduction could be greater than 50% (Fig. 3D).

Major vault protein (MVP), also known as lung resistance protein, is a major component of a ribonucleoprotein organelle called vault, which has been implicated in drug resistance in cancer cells^15^. The MVP protein (~110 kDa) was induced by DDP in a dose-dependent manner. In the presence of YPFS, the DDP-induced expression of MVP was markedly reduced (Fig. 3E). Similar to MVP protein, the DDP-induced MVP mRNA was suppressed by treatment with YPFS (Fig. 3F).

### YPFS increases DDP-induced ROS accumulation and MMP depletion

**Figure 4:**
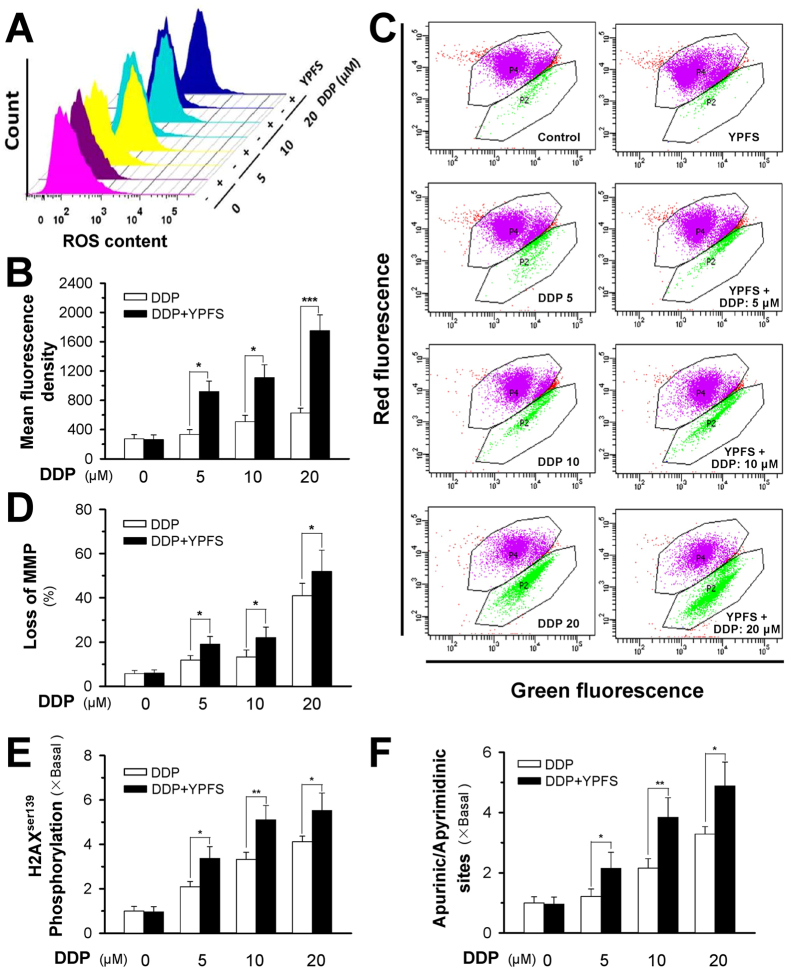
YPFS increases DDP-induced ROS formation, MMP loss and DNA damage in DPP-treated A549/DDP cells. (**A**) Cultured A549/DDP cells were treated with or without YPFS (1 mg/mL) for 12 hours, followed by 24 hours of DDP treatment at various concentrations. The amount of ROS was detected by a flow cytometry. (**B**) Mean fluorescence density of ROS level was calibrated from (**A**). (**C**) Cultured A549/DDP cells were treated with or without YPFS (1 mg/mL) as in (**A**). The MMP was detected by a flow cytometry. (**D**) The percentage of MMP loss was calibrated from (**C**). Values are in percentage of MMP loss as compared to control (no drug treatment). (**E**) Quantification of DNA damage by measuring H2AX^Ser139^ phosphorylation in A549/DDP cells. Cultured A549/DDP cells were treated with or without YPFS (1 mg/mL) for 12 hours, followed by 48 hours of DDP treatment at various concentrations. Values are relative amount in fold of change (X Basal) to control (no drug treatment). (**F**) DNA damage determined by quantification of apurinic/apyrimidinic sites. The cell treatment was done as in (**E**). Values are relative amount in fold of change (X Basal) to control (no drug treatment). Results are expressed as the mean ± SEM from three separate experiments, *n* = 3. *p < 0.05, **p < 0.01, or ***p < 0.001, versus DDP-treated alone.

The pro-apoptotic activities of reactive oxygen species (ROS) and mitochondrial membrane potential (MMP) were determined in DDP-treated A549/DDP cells. Using 2′,7′-dichlorofluorescin diacetate (DCFH-DA) and 5,5′,6,6′-Tetrachloro-1,1′,3,3′-tetraethylbenzimidazolocarbocyanine iodide (JC-1) based detection as well as flow cytometry, the accumulation of ROS and MMP were observed after treating A549/DDP cells with either DDP alone or in combination with YPFS pre-treatment. The formation of ROS in cultured A549/DDP cells was measured by flow cytometry, as generated by DCFH-DA. Application of DDP induced ROS accumulation in a dose-dependent manner (Fig. 4A,B). The ROS levels in A549 cells that were exposed to various concentrations of DDP were significantly higher than that of A549/DDP cells (Supplementary Fig. 4A,B). Similar to cell apoptosis, the DDP-induced ROS level was markedly increased with pre-treatment of YPFS; the difference was greater than 2-fold when treated with a high concentration of DDP (Fig. 4A,B), which implies that YPFS attenuates the decline of DDP-induced ROS formation in A549/DDP cells. In addition, MMP was detected by JC-1 in DDP-treated A549/DDP cells. The loss of MMP was increased with DDP application in a dose-dependent manner, which was further significantly enhanced with the pre-treatment of YPFS (Fig. 4C,D). The alteration of ROS and MMP levels could result in increased cell death triggered by the co-treatment of DDP and YPFS.

Due to the increase in DDP-induced ROS levels in the presence of YPFS, we hypothesized that YPFS plays a role in the DNA damage of cancer cells. DNA double-stranded breaks were quantified by measuring the phosphorylation of histone 2AX and the number of apurinic/apyrimidinic sites in the genomic DNA. DDP induced histone 2AX phosphorylation and apurinic/apyrimidinic sites in A549/DDP cells, which were notably lower than that in A549 cells (Supplementary Fig. 4C,D). In A549/DDP cells, YPFS significantly increased the level of histone 2AX phosphorylation and the number of apurinic/apyrimidinic sites, which indicated that the reduced DNA damage in A549/DDP cells could be sensitized by YPFS (Fig. 4E,F).

### YPFS modulates DDP-induced p62/TRAF6 signalling

**Figure 5:**
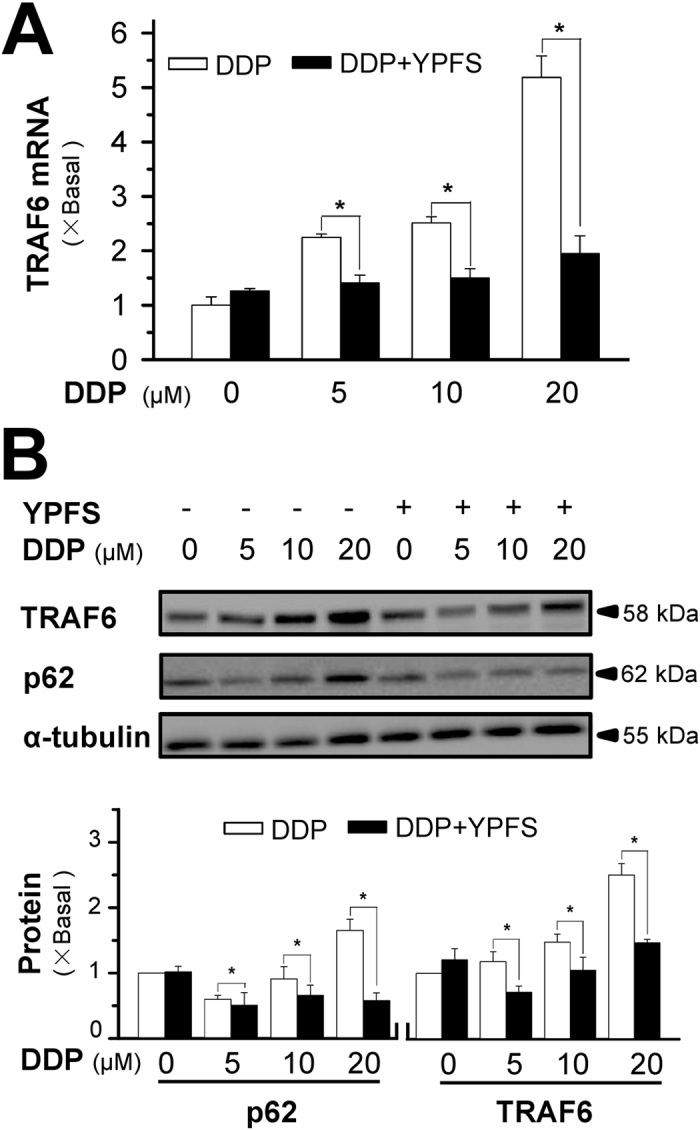
YPFS suppresses DDP-induced expression of p62/TRAF6 in A549/DDP cells. (**A**) A549/DDP cells were treated with or without YPFS (1 mg/mL) for 12 hours, followed by 48 hours of DDP treatment at various concentrations. The mRNA expression of TRAF6 was determined by real-time PCR. (**B**) The protein expressions of p62 (~62 kDa) and TRAF6 (~58 kDa) were determined by western blotting (upper panel). Expression of α-tubulin (~55 kDa) served as a control. The full length bot of p62 is supplied in supplementary data Fig. 6. The quantitation of p62 and TRAF6 expressions were calibrated (lower panel). Values are in fold of change (X Basal) to control (no drug treatment). Each point represents the mean ± SEM, *n* = 3. *p < 0.05 versus DDP-treated alone.

TNF receptor-associated factor 6 (TRAF6) is a ubiquitin ligase that regulates a diverse array of physiological processes and interacts with cytoplasmic protein p62. The protein-protein interacting modules enable the protein to serve as a scaffold for activation of transcription factor nuclear factor-κB (NF-κB)^16^, which subsequently controls tumour cell survival. Moreover, the p62/TRAF6 signalling was shown to correlate with enhanced ROS production, which may mediate the drug sensitivity of chemotherapy^17^,^18^. In cultured A549/DDP cells, DDP application induced the mRNA level of TRAF6 in a dose-dependent manner; however, pre-treatment with YPFS completely blocked the DDP-induced TRAF6 expression (Fig. 5A). In addition to the transcript expression, the amount of TRAF6 protein at ~58 kDa was increased with DDP treatment; this protein induction was also completely blocked by pre-treatment with YPFS (Fig. 5B). The level of p62 at ~62 kDa, an interacting partner of TRAF6, was also induced with treatment of DDP and was completely abolished by pre-treatment with YPFS (Fig. 5B).

**Figure 6:**
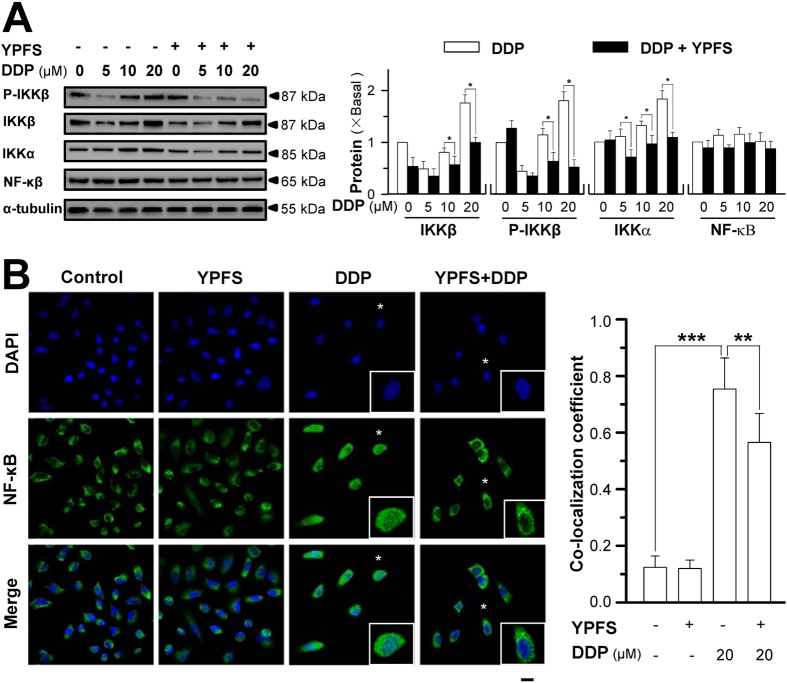
YPFS suppresses DDP-induced NF-κB activation in A549/DDP cells. (**A**) Cultured A549/DDP cells were treated with or without YPFS (1 mg/mL) for 12 hours, followed by 48 hours of DDP treatment at various concentrations. The amounts of phosphor-IKKβ (~87 kDa), IKKβ (~87 kDa), IKKα (~85 kDa) and NF-κB p65 subunit (~65 kDa) were determined by using specific antibodies (left panel). α-Tubulin (~55 kDa) served as a control. The band intensity was calibrated (right panel). Values are in fold of change (X Basal) to control (no drug treatment). (**B**) Immunofluorescence staining localization of NF-κB p65 by antibody, and nuclei staining by DAPI, in A549/DDP cells. Treatment was as that in (**A**), except 20 μM DDP was used. Asterisks indicate nuclei with NF-κB staining, also the enlarged cells in bottom right corner (left panel). Co-localization coefficients, co-localizing pixel for NF-κB p65 in channel 1 (Ch1) were calculated relative to the total number of pixels for the nuclei (T1) by using Zeiss co-localization coefficient function software (right panel). Bar = 20 μM. Results are expressed as the mean ± SEM from three separate experiments. *n* = 3. *p < 0.05, **p < 0.01, or ***p < 0.001.

The downstream effector of p62/TRAF6 signalling stimulated by YPFS and DDP treatment was identified. The expression of IκB kinase α (IKKα at ~85 kDa) and IκB kinase β (IKKβ at ~87 kDa) as well as phosphor-IKKβ (at ~87 kDa) was determined. DDP application induced the expression of IKKα and IKKβ as well as the phosphorylation of IKKβ in a dose-dependent manner, and these inductions were completely blocked by pre-treatment with YPFS (Fig. 6A). The expression of the NF-κB p65 subunit was not altered in the presence of DDP or YPFS (Fig. 6A). In addition, the translocation of NF-κB p65 in A549/DDP cells when co-treated with DDP and YPFS was determined using immunofluorescence staining (Fig. 6B). DDP at a concentration of 20 μM significantly induced the nuclear accumulation of NF-κB p65 by ~7-fold. YPFS alone did not alter the localization of NF-κB p65; however, YPFS co-treatment with DDP significantly reduced DDP-induced NF-κB p65 nuclear accumulation (Fig. 6B), suggesting that the downstream signalling of p62/TRAF6 was regulated by YPFS in DDP-induced multi-drug resistance.

### YPFS increases the efficacy of DDP in mouse xenograft models

**Figure 7:**
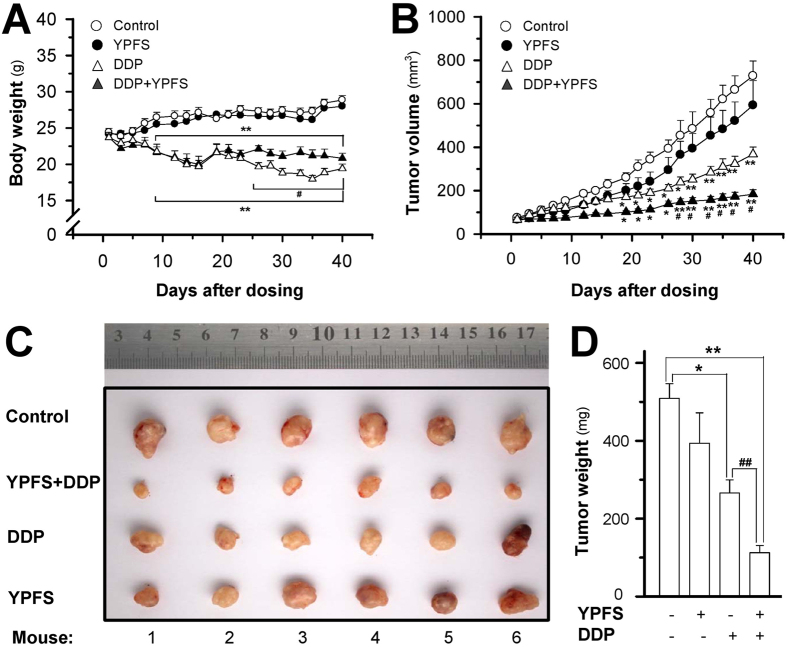
YPFS increases anti-tumor activity of DDP in A549/DDP bearing nude mice. Lung cancer was induced in nude mice by implanting A549/DDP lung cancer cells subcutaneously in the flank of mice. The tumor was allowed to grow at ~80 mm^3^. Thereafter, DDP (3 mg/kg) in the absence, or presence of YPFS (4 g/kg), was administered. (**A**) The body weight was checked 2–3 times per week over a period of 40 days. (**B**) The mean tumor volume in each group after drug treatment. (**C**) Tumors excised out of the mice at day 40. (**D**) Mean tumor weight in each group at the end of treatment. Data represents Mean ± SEM of six mice per group. *p < 0.05 and **p < 0.01 vs control; ^#^*p* < 0.05 and ^##^*p* < 0.01 vs DDP-treated group.

To further explore the sensitizing effect of YPFS in DDP-treated tumour growth, we employed an ectopic implantation model in nude mice. The body weight of mice was decreased after DDP treatment, and the weight reduction was improved by co-treatment with DDP and YPFS (Fig. 7A). In control mice, the tumour volume gradually increased in a time-dependent manner (Fig. 7B). Treatment with DDP significantly suppressed the tumour volume, and the volume reduction was more robust when co-treated with DDP and YPFS (Fig. 7B). YPFS treatment alone did not show a significant effect on the tumour volume. After 40 days of treatment, tumours were removed from sacrificed mice and were weighed. Similarly, a significant difference was observed in the tumour weights of the control and DDP-treated animals, and again, a synergistic effect was observed in the YPFS and DDP co-treated animals (Fig. 7C,D). Compared to the control tumour-bearing mice, co-treatment with DDP and YPFS reduced the tumour weight by greater than 80% (Fig. 7D). The tumour inhibitory rate (IR) was compared in different treatments. The IR was calculated as follows: IR (%) = (1 − TWt/TWc) × 100, where TWt and TWc are the mean tumour weight of treated and control groups, respectively. The IRs of the DDP, YPFS and combination treatments were 47.7%, 19.6%, and 77.9%, respectively. Thus, the IR of co-treatment with DDP and YPFS was significantly increased when compared with DDP treatment alone. These results indicated that YPFS could notably improve the DDP-suppressed cancer effect and reduce the toxicity generated by DDP during cancer treatment.

## Discussion

Here, we investigated the anti-drug resistance effect of YPFS on the DDP-resistant lung cancer cell line A549/DDP. The inhibition of cell growth induced by DDP was significantly enhanced by co-treatment with YPFS. Similarly, the rate of apoptosis of A549/DDP cells was increased by the co-treatment with DDP and YPFS, which was confirmed by the increased levels of apoptotic markers, e.g., cleaved-PARP, cleaved caspase 3 and cleaved caspase 9. A final verification of the effects of DDP and YPFS co-treatment was demonstrated by a robust reduction of tumour size in lung cancer-bearing mice. The role of YPFS in DDP-treated A549/DDP cells could consist of several factors: (i) it increases the intracellular DDP concentration; (ii) it suppresses the expression of drug transporters; (iii) it increases the ROS level and loss of MMP; (iv) it suppresses the expression of MRP2 and MVP; and (v) it suppresses the p62/TRAF6 signalling and the activation of the downstream protein NF-κB. The final outcome of YPFS treatment was an increase in the sensitivity of DDP-induced toxicity in A549/DDP cells as well as a decrease in the tumour size in mice. This is the first time that the reversing effect of YPFS in DDP-induced resistance has been investigated both *in vitro* and *in vivo*.

Decreased intracellular accumulation of DDP is one of the predominant mechanisms by which lung cancer cells elude the toxicity of DDP. After treatment with YPFS, the intracellular level of DDP in cancer cells was markedly increased. This increase could be triggered by YPFS via the following two mechanisms: (i) by blocking the activities of efflux transporters and (ii) by reducing the expression of transporters. The direct blockage of the efflux transporters could be supported by an increase of DDP within the cells during a short exposure time as well as the inhibition of the efflux transporter ATPase, as previously reported^19^. Another mechanism by which YPFS treatment could increase DDP levels in the cells is through suppressing the DDP-induced expression of efflux transporters; this reduction of transporters was illustrated here, i.e., the reduced expression of MRP1, MRP2, MRP3 and BCRP, which was highly related to the DDP resistance by increasing the export of DDP^20^. Among these efflux transporters, MRP2 is the major ATPase that contributes to DDP efflux in the resistant cells^21^,^22^. Here, the expression of MRP2 induced by DDP was much higher when compared with the other transporters, and this result was consistent with previous reports^21^,^22^. YPFS significantly attenuated the increase of the transporters MRP1, MRP2, MRP3 and BCRP, with the most notable change in MRP2, which suggested the outcome of YPFS treatment occurs through the increase of the intracellular DDP concentration. Moreover, the expression of MVP was notably suppressed by YPFS. MVP is proposed to be involved in intracellular drug distribution, which plays a role in the extrusion of chemotherapy drugs from the nucleus^23^. Overall, DDP levels were increased within the cells in the presence of YPFS.

The intracellular concentration of DDP induces the redox resetting, which contributes to the high levels of ROS and ROS-scavenging systems to cope with the exogenous stress in drug-resistant malignant cells^24^. ROS plays a central role in DDP-induced cell death^9^, which can be rescued by the ROS scavenger N-acetyl-L-cysteine (Supplementary Fig. 5). Additionally, increased ROS makes a conformational change in adenine nucleotide translocase (ANT), leading to a mitochondrial permeability transition through pore opening and ultimately a change in MMP. High levels of MMP are usually observed in cancer cells, especially in lung cancer, e.g., A549 and H446 cell lines^25^,^26^. Similarly, the high levels of MMP possess a stronger resistance to the apoptotic inducers. The abrogation of the altered redox status, e.g., the elevated ROS level, could ultimately determine the cell fate towards apoptotic or necrotic cell death^27^. The main consequence of elevated ROS is DNA damage, which is the main mechanism that is responsible for DDP-induced cell death. Because resistant cancer cells can trigger antioxidant-signalling pathways to reduce the DNA damage caused by ROS, we wanted to find out whether the combination of YPFS and DDP could increase the DDP-induced DNA damage^28^. DDP stimulates H2AX phosphorylation on serine 139 (γH2AX). The formation of γH2AX is one of the earliest chromatin modification events in DNA damage, which is important for the coordination of the DNA repair activities^29^. The apurinic/apyrimidinic (AP) sites are one of the major types of DNA lesions that are formed as intermediates during base excision and oxidized base repair. Co-treatment with YPFS and DDP significantly increased the aforementioned parameters during DNA damage. The level of DNA damage in resistant cells when co-treated with YPSF and DDP was similar to that induced by treatment with DDP alone in non-resistant cells, which indicated that DDP-induced DNA damage in A549/DDP cells was sensitized by YPFS treatment.

Here, YPFS treatment enhancement of DDP-induced ROS and MMP depletion could be a direct or an indirect effect. First, YPFS alone did not show any effect on the ROS and MMP levels, which is the opposite notion of a direct effect. Therefore, the indirect effect of YPFS could be an outcome of increased intracellular DDP levels, which subsequently caused further changes in the ROS and MMP levels. However, this hypothesis needs further investigation.

Activation of the NF-κB canonical pathway is one of the strategies by which cancer cells become resistant to increased ROS levels, ultimately leading to the translocation of NF-κB into the nucleus and resulting in the transcription of pro-survival genes^30^. However, a decrease in NF-κB translocation was observed when the cells were co-treated with YPFS and DDP, instead of an increase in the translocation level that might be triggered by ROS. Thus, we hypothesize that other signalling pathways induced by the co-treatment of YPFS and DDP may influence the activation of NF-κB, shift the modulation balance and result in reduced activity. Signalling of p62/TRAF6 has been proposed to play a role in the development of carcinomas. Several studies have revealed that p62 is overexpressed in lung carcinoma cells, especially in A549 cells^31^,^32^,^33^. The binding of p62 with TRAF6 leads to the phosphorylation of IKKβ and, subsequently, to the activation of NF-κB. The upregulated p62/TRAF6 signalling promotes cancer cell survival^33^. The induction of this signalling by DDP could partly account for the drug-resistance of A549/DDP cells. The mechanism by which YPFS suppresses DDP-activated p62/TRAF6 signalling, however, has not been elucidated, but this suppression could be an indirect effect. YPFS treatment alone did not show any effect on p62/TRAF6 signalling, suggesting that the role of YPFS requires DDP activation first. Possibly, YPFS could regulate the feedback control of DDP-induced signalling.

Studies in the A549/DDP xenograft nude mice further proved the sensitized function of YPFS on the DDP-induced anti-cancer effect. The *in vivo* studies showed that the combination of YPFS and DDP displayed a notably reduced growth rate and tumour volume when compared with the treatment of DDP alone. Additionally, body weight was significantly higher when combined with YPFS treatment. These data indicated that the anti-cancer effect of DDP was improved by YPFS treatment with less toxicity.

Chinese herbal medicine could be a rich source to search for efflux transport inhibitors. Supporting this notion, the San Geng herbal decoction was shown to downregulate the expression of P-gp, and similarly, Si Wu Tang reversed doxorubicin multi-drug resistance^34^. Thus, mechanistic studies are needed to identify the ingredients in Chinese herbs that are agents for multi-drug resistance. Although the exact ingredients within YPFS that exhibit the anti-drug resistance have not been elucidated, we hypothesize that the flavonoidic compounds, found abundantly in YPFS, could possibly be the targeted chemicals. Flavonoids have been found to modulate the transporter-mediated drug efflux^35^, which could inhibit the efflux transporter ATPase by interacting directly with the ATP-binding site^36^. In YPFS treatment, calycosin and formononetin are the major flavonoids derived from AR^19^, which have been shown to affect the efflux transporters in cultured intestinal cells^14^. However, the mechanisms involved in this process need further research.

YPFS has been used clinically for more than six thousand years, which demonstrates its safety in clinical treatment. Our study first illustrated the effect of YPFS treatment in mediating the chemo-sensitivity of DDP, which is the most common chemotherapy drug for lung cancer, especially for advance lung cancer patients. Historically, the clinical application of YPFS has mainly focused on immunological modulation. These findings, however, will aid in the novel application of YPFS in cancer patients, as well as the strategy for reversing DDP-induced drug resistance during lung cancer treatment and will be more applicable because of its safety when compared with chemosensitizers.

## Materials and Methods

### Reagents and antibodies

The FITC-labelled Annexin V Apoptosis Detection Kit was obtained from BD Biosciences (San Jose, CA, USA). DNA Damage Quantification Colorimetric Kit was purchased from Biovision (Milpitas, CA, USA) and EpiQuik *In Situ* DNA Damage Assay kit was obtained from Epigentek (Farmingdale, NY, USA). JC-1, cisplatin, and DCFH-DA were purchased from Sigma-Aldrich (St. Louis, MO, USA). The culture medium was obtained from Invitrogen Technologies (Carlsbad, CA, USA). The antibodies were obtained from the following sources: TRAF6 and MVP from Abcam (Cambridge, UK); α-tubulin from Sigma-Aldrich; p62, NFκB, cleaved-PARP, cleaved-caspase 3, cleaved-caspase 7, cleaved-caspase 9, IKKβ, IKKα, phosphor-IKKβ, horseradish peroxidase (HRP)-conjugated goat anti-rabbit antibody, HRP-conjugated goat anti-mouse antibody, and Alexa Fluor 488-conjugated goat anti-mouse antibody from Cell Signalling Technology (Danvers, MA, USA).

### Plant material and herbal preparation

The roots of *A*. *membranaceus* var. *mongholicus* (AR), the rhizomes of *A*. *macrocephala* (AMR) and the roots of *S*. *divaricata* (SR) were collected from the Shanxi province, Anhui province and Heilongjiang province, respectively. The plant materials were authenticated by one of the authors, Dr. Tina Dong, and the herbal specimens were deposited at the Center for Chinese Medicine at The Hong Kong University of Science and Technology. The YPFS herbal extracts were prepared according to the method previously described^37^. Briefly, the herbal mixture (AR: AMR: SR in a 1:2:1 weight ratio) was boiled in 8 volumes of water (v/w) by moderate heating for 2 hours. The residues were then re-boiled in 6 volumes of water for 1 hour. The extracts, which were pooled from two extractions, were filtered, dried by lyophilization and stored at 4 °C.

### Cell viability assay

The A549 cell line was obtained from the American Tissue and Cell Collection (ATCC). The A549/DDP (DDP multi-drug resistance) cell line was purchased from the China Academy of Military Medical Science (Beijing, China). Cells were cultured in RPMI 1640 with 10% foetal bovine serum, 1% penicillin and 1% streptomycin. The cells were maintained in a humidified 5% CO_2_ atmosphere at 37 °C. The cells were trypsinized and plated in 96-well plates at 3,000 cells per well. The cells were treated with various concentrations of DDP in the presence or absence of YPFS (1 mg/mL) for 48 hours after settling to the base. MTT[3-(4, 5-dimethyl thiazol-2yl)-2, 5-diphenyltetrazolium bromide] (100 μg/well) was added to each well and incubated for an additional 4 hours at 37 °C in 5% CO_2_. After the removal of the medium, the formazan crystals formed were dissolved in 150 μL of 100% dimethylsulfoxide (DMSO) per well by mixing on a shaker for 15 min, and then the spectrophotometric absorbance at 570 nm was determined. The Combination Index (CI) was calculated using CompuSyn software (www.combosyn.com). The synergy-level classifications were assigned as described in the CompuSyn manual. CI values that are <1, 1 and >1 indicate a synergism, additive effect and antagonism, respectively.

### Apoptosis detection

A549/DDP cells were seeded in 35-mm tissue-culture plates (10 × 10^4 ^cells/well) and allowed to grow for 24 hours in culture medium before the treatment. The apoptosis assay was conducted using the Annexin V-FITC/PI Apoptosis Detection kit by flow cytometry (BD Biosciences) following the manufacturer’s instructions. Briefly, both floating and adherent cells were collected and washed in phosphate-buffered saline (PBS). Cells at 10^5^ were incubated in 100 μL of binding buffer, containing annexin-V/FITC and propidium iodide, for 15 min at room temperature in the dark. The samples were automatically acquired using the loader with the acquisition criteria of 10,000 events for each tube, and the quadrants were set according to the population of viable, unstained cells in untreated samples. The data were analysed using FACSAria equipped with the CellQuest Software (BD Biosciences)^38^.

### Detection of intracellular DDP

Cells were treated with 100 μM DDP in the absence or presence of YPFS for 6 hours. The cells were then harvested and washed several times with PBS. The cell suspension was centrifuged, the pellets were then mineralized in 500 μL 70% HNO_3_ and spiked with 40 μg/L cadmium at 80 °C overnight, and then diluted with 2.5 mL H_2_O. Platinum determination was performed using inductively coupled plasma optical emission spectrometry (ICP-OES)^39^.

### Measurement of intracellular ROS

A549/DDP cells were treated with YPFS for 12 hours, followed by 24 hours of DDP treatment at various concentrations. Control cells and drug-treated cells were washed twice in PBS and then incubated with DCFH-DA (15 μM) at 37 °C for 30 min. After washing twice in PBS, cells were collected by trypsin. Cells were analysed using a flow cytometer, and ROS was measured as the mean fluorescence intensity. The results were analysed with Flowjo v7.6 software^40^.

### Measurement of MMP

A549/DDP cells were treated with YPFS for 12 hours, which was followed by 24 hours of DDP treatment at various concentrations. The cells were collected and washed in PBS and then were incubated with 1 mL JC-1 staining solution (5 *μ*g/mL) for 30 min. Subsequently, the cells were measured using flow cytometry. The fluorescence generated by JC-1, a J-aggregated form, was measured at the excitation wavelength of 561 nm and the emission wavelength of 582 ± 25 nm. The fluorescence of the JC-1 monomer was detected at the excitation wavelength of 488 nm and the emission wavelength of 530 ± 30 nm. The results were analysed using the FlowJo v7.6 software^41^.

### RNA isolation and real-time PCR

Total RNAs were extracted using the RNAzol RT reagent and were reversed transcribed into cDNAs, as previously described^42^. Briefly, the cells were collected and lysed in 1 mL RNAzol RT reagent. The lysate was added to 0.4 mL distilled water, vortexed vigorously for 15 sec, stored for 15 min at room temperature, and then centrifuged at 12,000  × *g* for 15 min at 4 °C. The aqueous layer was collected and added to 0.4 mL 75% ethanol, stored for 10 min for RNA precipitation, and followed by centrifugation at 12,000  × *g* for 5 min. The RNA pellets were then washed twice with 0.4 mL 75% ethanol, dried, re-suspended in RNAase free water, and then quantified by spectrometry. One μg of total RNA was reverse transcribed by MMLV according to the manufacturer’s instructions. Real-time PCR was performed in 96-well plates using 100 ng of cDNA input and the SYBR Green Master mix (Roche). The following primers were used: 5′-CCG TGG AGA GGC TCA AGG AGT ATT C-3′ (S) and 5′-GAT GAT GGT GAT CTT GAA GCG GAG G-3′ (AS) for MRP1; 5′-CTC CTG CCT GTT CTT CAT CTC CTA C-3′ (S) and 5′-CTT CAT CAA CTT CCC AGA CAT CCT C-3′ (AS) for MRP2; 5′-TTC CGC TTC ACC ACC TTC TAC AT-3′ (S) and 5′-GTC TGC TTT TCC TGC TTC CTC CAT-3′ (AS) for MRP3; 5′-TCT GGA GAT GTT CTG ATA AAT GGA G-3′ (S) and 5′-CAG ACC TAA CTC TTG AAT GAC CCT G-3′ (AS) for BCRP; 5′-TTT GCT CTT ATG GAT TGT CCC C-3′ (S) and 5′-CAT TGA TGC AGC ACA GTT GTC-3′ (AS) for TRAF6; 5′-GTC TTC GGG CCT GAG CTG GTG TCG-3′ (S) and 5′-CTT GGC CGT CTC TTG GGG GTC CTT-3′ (AS) for MVP; and 5′-AAC GGA TTT GGC CGT ATT GG-3′ (S) and 5′-CTT CCC GTT CAG CTC TGG G-3′ (AS) for GAPDH. The SYBR Green signal was detected using the Mx3000ptm multiplex quantitative PCR machine from Stratagene (La Jolla, CA). The data were normalized to the amount of the GAPDH housekeeping genes.

### Western blot analysis

After treatment, the cells were rinsed with cold PBS and harvested in a low-salt lysis buffer, containing 100 mM HEPES, 150 mM NaCl, 1 mM EGTA, 1 mM EDTA, 1% Triton, 1% NP-40, 3 mM benzamidine, 10 mM NaF, 1 mM Na_3_VO_4_, and 20 μg/mL each of aprotinin and leupeptin (pH 7.5). The collected cell lysates were vortexed for 10 min, and the insoluble cell debris were removed using centrifugation. The total protein concentrations were measured using the Bradford method^43^, and then all lysates were diluted to the same concentration. The cell lysates were boiled in a gel-loading buffer (20% glycerol, 10% β-mercaptoethanol, 6% SDS, 125 mM Tris–HCl, pH 6.8, 0.005% bromophenol blue; 1:5) at 95 °C for 10 min. The protein extracts were resolved in an 8 or 15% acrylamide gel by electrophoresis and then transferred to nitrocellulose membranes. Membranes were blocked with 5% milk in a Tris-buffered saline containing 0.1% Tween-20 (TBST) for 1 hour before an overnight incubation at 4 °C with various primary antibodies, typically at a 1:1000 dilution. Blots were rinsed with TBST and incubated for 2 hours at room temperature with HRP-conjugated secondary antibody at a 1:5,000 dilution. Reactive bands were visualized by the Chemidoc Imaging System (Bio-Rad; Hercules, CA) using ECL (Invitrogen). The intensities of the immune-reactive bands were calculated in the Chemidoc Imaging System.

### DNA damage

DNA damage was assessed by quantification of the apurinic/apyrimidinic sites (BioVision,CA,USA) and the γH2AX assay. Cells were pre-treated with YPFS for 12 hours, followed by treatment with DDP at various concentrations for an additional 48 hours. For the AP sites detection, genomic DNA was extracted. The number of AP sites in the DNA was detected by DNA Damage Quantification Colorimetric Kit following the manufacturer’s instructions. For the γH2AX assay, the double-stranded breaks were detected using the EpiQuik *In Situ* DNA Damage Assay kit (Epigentek, Farmingdale, NY, USA) according to the manufacturer’s instructions. The absorbance signal was normalized to the cell number in each sample, and the samples were calculated relative to the untreated control^44^,^45^.

### Immunofluorescence

A549/DDP cells were placed on glass coverslips in 12-well plates. The cells were treated with 20 μΜ DDP in the presence or absence of YPFS (1 mg/mL) for 48 hours after settling to the base. The cells were washed twice with PBS. After aspirating the medium, the cells were fixed for 15 min at room temperature with 4% formaldehyde in 1X PBS. The cells were then incubated for 60 minutes in the blocking buffer (1X PBS/5% BSA/0.3% Triton™ X-100). Fifty μL of diluted primary antibody (1:800) was applied to each coverslip and incubated overnight at 4 °C. After washing in 1X PBS, the cells were incubated with Alexa Fluor 488-conjugated goat anti-mouse secondary antibody at a 1:500 dilution for 2 hours at room temperature in the dark and followed by DAPI nuclear staining (5 mg/mL) for 15 min. After washing, the images were taken using a Zeiss Laser Scanning Confocal Microscope (LSM7 DUO). To analyse the extent of the nuclear translocation of the NF-κB p65 subunit, the co-localization coefficient (Ch1-T1) for the nuclei occupied by the NF-κB p65 subunit was calculated from the merged images using the LSM ZEN 2009 image analysis program. The co-localization coefficients (for NF-κB p65 co-localization in the nuclei) were calculated using the Zeiss co-localization coefficient function software, in which all background pixels were considered, and the number of co-localizing pixels (for NF-κB p65) in channel 1 (Ch1) was calculated relative to the total number of pixels (for the nuclei).

### Animal xenograft studies

Five-week old male BALB/C nu/nu nude mice, weight of 17–18 g, were obtained from Shanghai SLAC Laboratory Animal Company (Shanghai, China). The nude mice were maintained in pathogen-free conditions at 22 °C ± 2 °C, at 70% relative humidity and under a 12-hour light/dark cycle. A549/DDP cells (1 × 10^7^/0.2 ml PBS) were harvested and subcutaneously implanted into the left flank of nude mice. The tumours were harvested after they reached a size greater than 600 mm^3^ and then were cut into small pieces with approximately equal sizes (Ф = 0.5–1.5 mm). The tumour pieces were transplanted subcutaneously into the left flanks of nude mice. On day 21 post tumour implantation, the mice were randomized into four groups (each group consisting of six mice) according to their tumour volume so that all of the groups had a similar starting mean tumour volume. DDP (3 mg/kg) was administered 2–3 times per week by intraperitoneal injection, while the YPFS extract (4 g/kg) was administered intragastrically once per day. The dosage used in the mice was converted based on the surface area using the following formula: animal dose (g/kg) = HED (g/kg) × human *Km*/animal *Km*^46^. The YPFS human dose of 0.67 g/kg is equated to the mouse dose of 8.2 g/kg. The extraction of YPFS was ~50%; thus, the dose for the YPFS extract used in the animal study was 4 g/kg. The combination group was administered with both DDP and YPFS. Tumour size was measured 3 times per week using a caliper. The tumour volume (in cubic millimetres) was calculated using the ellipsoid formula: (D × (d^2^))/2, where ‘D’ represents the large diameter of the tumour and ‘d’ represents the small diameter. The nude mice were sacrificed after 40 days of treatment, and the tumour weights were recorded. All animal experiments were performed according to the National Institutes of Health Guide for the Care and Use of Laboratory Animals. The protocol was approved by Hangzhou Hibio Experimental Animal Ethics Committee (Permit Number: HB201510024) and under the guidelines of the “Principles of Laboratory Animal Care” (NIH publication No. 80-23, revised 1996) and Institutional Animal Care and Use Committees protocol (HBFM3.68-2015).

### Statistical analysis

Statistical comparisons were performed using one-way analysis of variance (ANOVA) followed by a Bonferroni multiple comparisons test using the SPSS 16.0 software. Data were expressed as the mean ± standard error of the mean (SEM). The statistical significance was taken at *p* < 0.05.

## Additional Information

**How to cite this article**: Lou, J.-S. *et al*. Yu Ping Feng San reverses cisplatin-induced multi-drug resistance in lung cancer cells via regulating drug transporters and p62/TRAF6 signaling. *Sci. Rep.* **6**, 31926; doi: 10.1038/srep31926 (2016).

## Supplementary Material

Supplementary Information
