# Supplementary material for: Yu Ping Feng San reverses cisplatin-induced multi-drug resistance in lung cancer cells via regulating drug transporters and p62/TRAF6 signalling
Source: Sci Rep. 2016 Aug 25;6:31926. doi: 10.1038/srep31926 (PMC4997265; doi:10.1038/srep31926)
Supplement: Supplementary Information [file srep31926-s1.pdf]

Yu Ping Feng San reverses cisplatin-induced multi-drug resistance in lung cancer cells via regulating drug transporters and p62/TRAF6 signaling

Jian-shu Lou[1,2], Lu Yan[1], Cathy W. C. Bi[1,2], Gallant K.L. Chan[1], Qi-Yun Wu[1], Yun-Le Liu[1], Yun Huang[1], Ping Yao[1], Crystal Y.Q. Du[3], Tina T.X. Dong[1], Karl W.K. Tsim*

[1]Division of Life Science, Center for Chinese Medicine, The Hong Kong University of Science and Technology, Clear Water Bay, Hong Kong, China

[2]Shenzhen Research Institute, The Hong Kong University of Science and Technology, Shenzhen, 518057, China

[3]Department of Biology, Hanshan Normal University, Chaozhou, Guangdong 521041, China

* Correspondence

Prof. Karl W.K. Tsim

Division of Life Science, and Center for Chinese Medicine, The Hong Kong University of Science and Technology, Clear Water Bay Road, Hong Kong, China

Phone: +852 2358 7332

Fax: +852 2358 1559

botsim@ust.hk

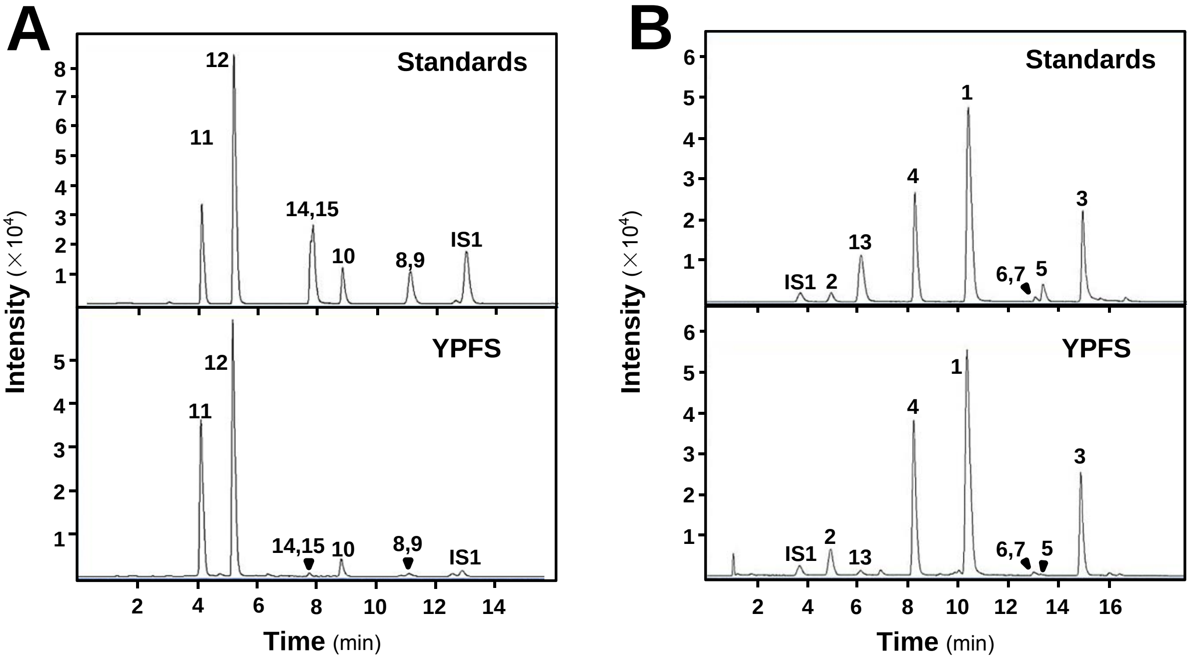

Supplementary Figure 1. Typical RRLC-QQQ-MS/MS chromatograms of 15 chemical markers in YPFS.

(A): The identification of prim-O-glucosylcimifugin (11), 5-O-methylvisammioside (12), psoralen (14), isopsoralen (15), atractylenolide III (10), atractylenolide II (9), atractylenolide I (8), crytotanshinone (IS1, internal standard 1) was made by a MS detector in the positive mode. **(B):** The identification of calycosin-7-O-β-D-glucoside (2), scopoletin (13), ononin (4), calycosin (1), astragaloside III (6), astragaloside IV (7), astragaloside II (5), formononetin (3), and aesculetin (IS2, internal standard 2) were made by a MS detector in the negative mode. Representative chromatograms are shown, *n* = 3.

Supplementary data

Fig. 1

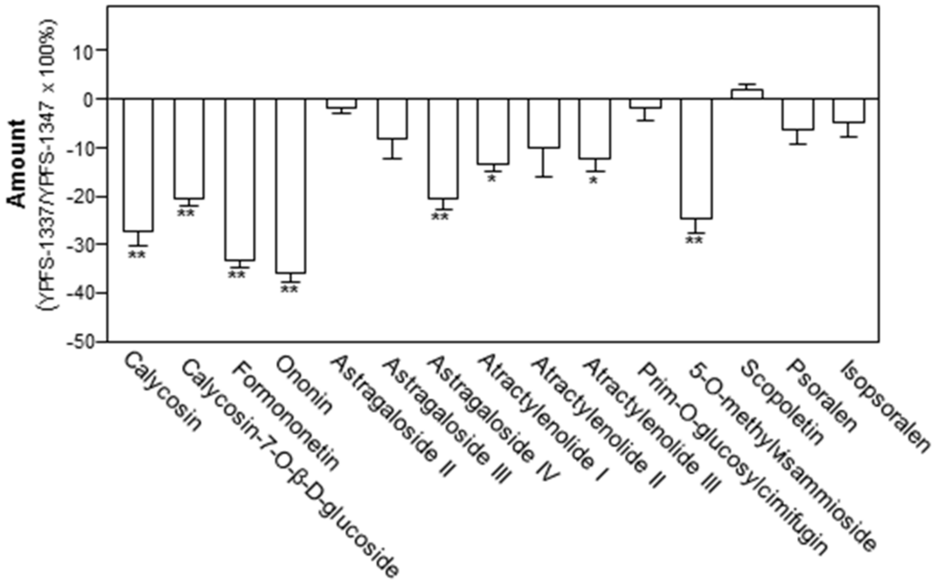

Supplementary Figure 2. Chemical comparison of different YPFS formulae.

The selected 15 analytes were determined in the water extracts of different YPFS formulae. The amounts of 15 analystes in YPFS-1337, prescribed by Wei Yilin, are expressed as % of YPFS-1347 (i.e. short form as YPFS as being analyzed here), prescribed by Zhu Danxi, where *n* = 3.

Supplementary data

Fig. 2

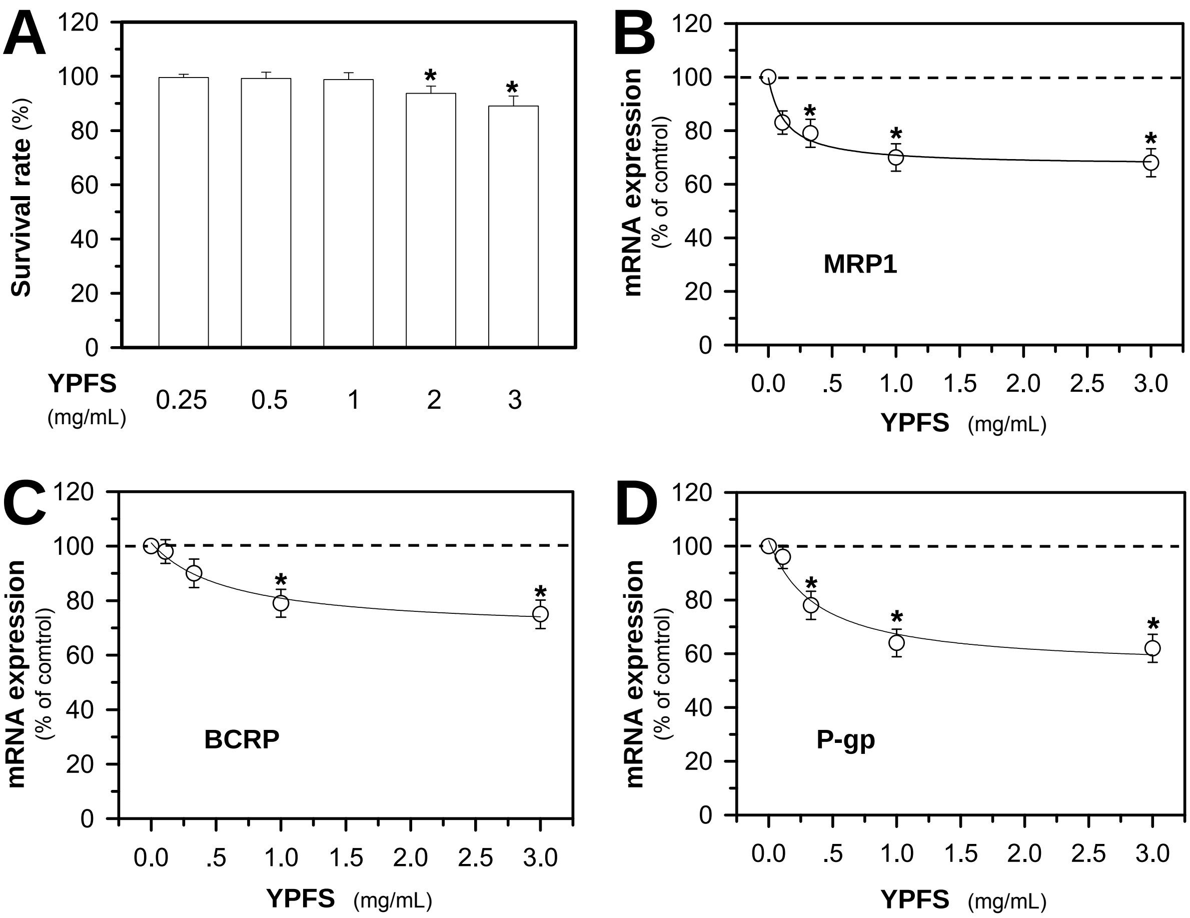

Supplementary Figure 3. The cell survival and mRNA expression of transporters under YPFS.

(A): A549/DDP cells, seeded in 96-well plates (3 X 10^3 cells/well), were allowed to adhere overnight, and subsequently which were treated with increasing concentration of DDP combined with YPFS (1 mg/mL) for 48 hours. Values are in percentage of cell growth inhibition. **(B):** The mRNA expression of MRP1. **(C):** The mRNA expression of BCRP. **(D):** The mRNA expression of P-gp. Cultured A549 cells were treated with the herbal extracts for 24 hours. The expression levels of MRP1,BCRP, and P-gp were revealed by real time PCR. GAPDH was used as an internal control for normalization in RT-PCR. Each point represents the Mean ± SEM, n = 3. *p < 0.05 versus control.

Supplementary data

Fig. 3

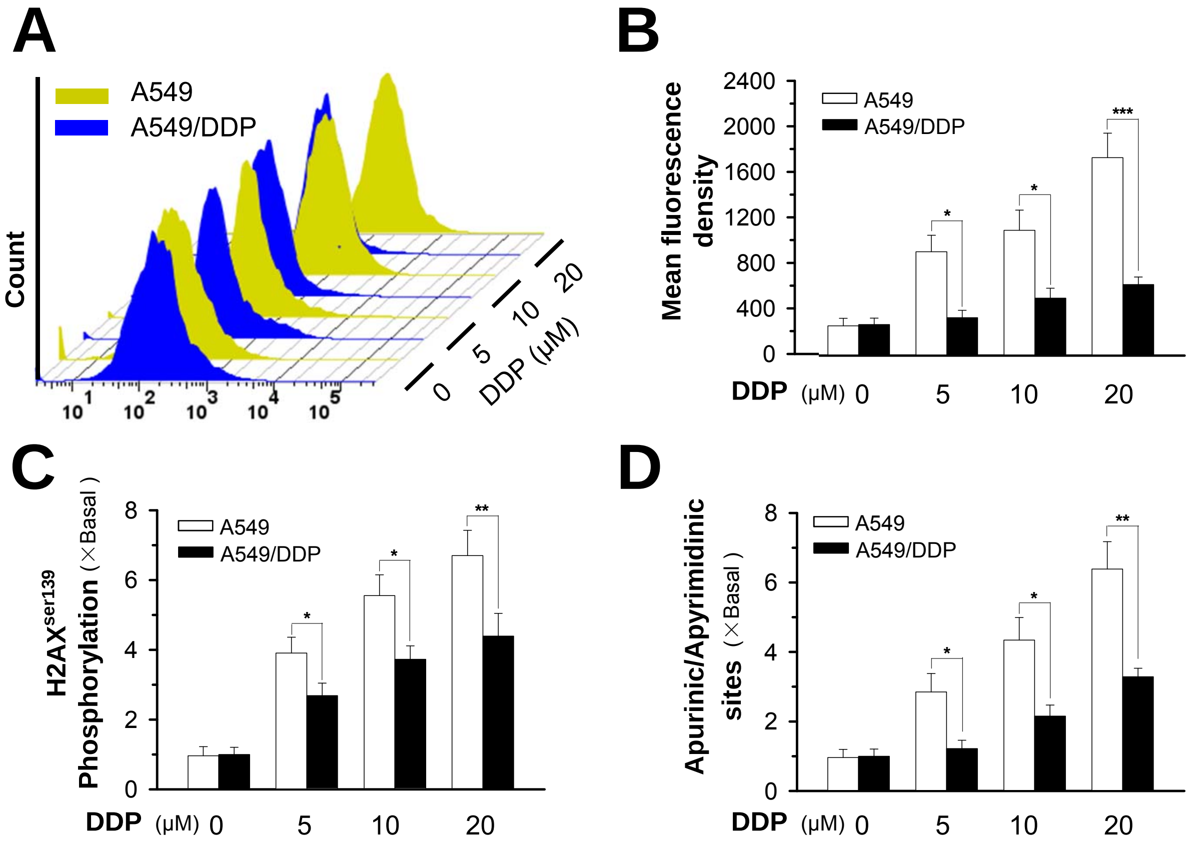

Supplementary Figure 4. DDP induces ROS formation and DNA damage on A549 and A549/DDP cell lines.

(A): Cultured A549/DDP and A549 cells were treated with or without YPFS (1 mg/mL) for 12 hours, followed by 24 hours of DDP treatment at various concentrations. The amount of ROS was detected by a flow cytometry. **(B):** Mean fluorescence density of ROS level was calibrated from (A). **(C):** Quantification of DNA damage by measuring H2AXSer139 phosphorylation in the A549/DDP and A549 cells. Cultured A549/DDP cells were treated with or without YPFS (1 mg/mL) for 12 hours, followed by 48 hours of DDP treatment at various concentrations. Values are relative amount in fold of change (X Basal) to control (no drug treatment). **(D):** DNA damage determined by quantification of AP sites. The treatment was done as in (C). Values are relative amount in fold of change (X Basal) to control (no drug treatment).Results are expressed as the Mean ± SEM from three separate experiments, $n = 3$. $^*p < 0.05$, or $^{**}p < 0.01$.

Supplementary data

Fig. 4

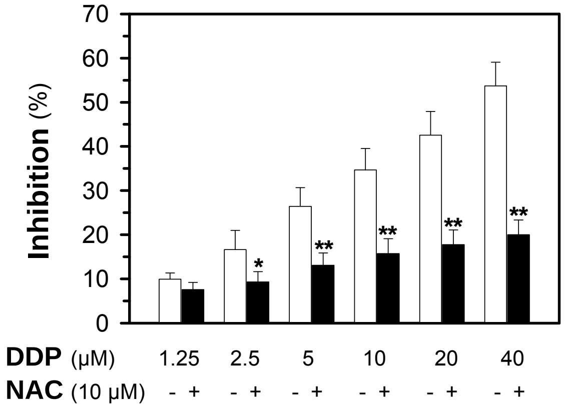

Supplementary Figure 5. The DDP-induced toxicity on A549/DDP cell is rescued by NAC.

A549/DDP cells, seeded in 96-well plates (3 X 10^3 cells/well), were allowed to adhere overnight and subsequently were treated with increasing concentration of DDP in the absence or presence of 30 min pre-treatment of 5 mM N-acetyl-L-cysteine (NAC) for 48 hours. Values are in percentage of cell growth inhibition. Each point represents the Mean ± SEM, $n = 3$. *$p < 0.05$, **$p < 0.01$ versus DDP-treated alone.

Supplementary data

Fig. 5

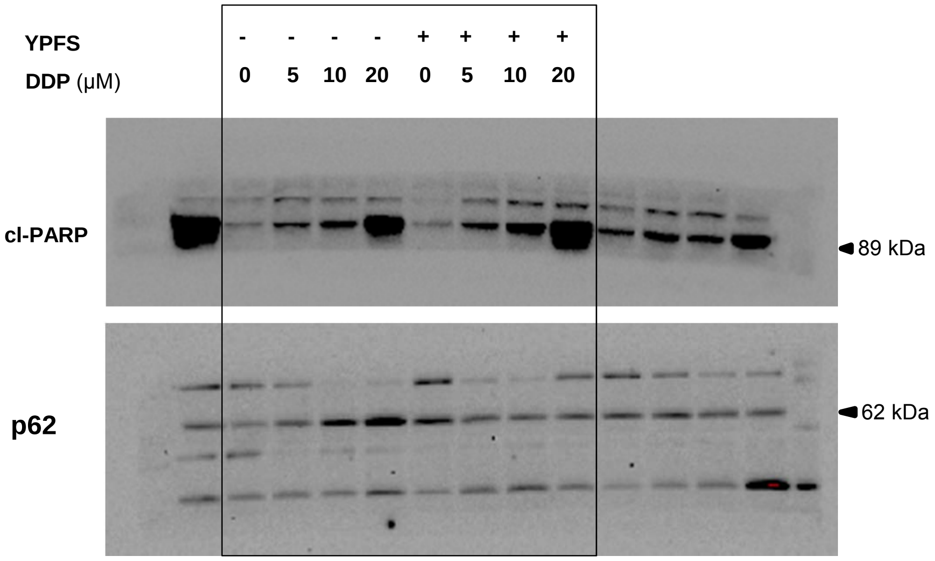

Supplementary Figure 6. The full-length blots of cl-PARP and p62 expression.

Cultured A549/DDP cells were treated with DDP at different doses for 48 hours in the presence or absence of YPFS (1 mg/mL; 12 hours of pre-treatment). Western blot analyses of cleaved (cl)-PARP at ~89 kDa and p62 at ~62 kDa .

Supplementary data

Fig. 6
